# Effects of transcranial direct current stimulation on patients with post-stroke fatigue: a study protocol for a double-blind randomized controlled trial

**DOI:** 10.1186/s13063-022-06128-9

**Published:** 2022-03-05

**Authors:** Xing Sun, Xiangli Dong, Qin Yuan, Guohua Yu, Lang Shuai, Chaolin Ma, Weiming Sun

**Affiliations:** 1 Department of Rehabilitation Medicine, The First Affiliated Hospital of Nanchang University, Nanchang, 330006 China; 2 First Clinical Medical School, Nanchang University, Nanchang, 330031 China; 3 Department of Psychosomatic Medicine, The Second Affiliated Hospital of Nanchang, Nanchang, 330006 China; 4 Department of Psychology, Jiangxi University of Traditional Chinese Medicine, Nanchang, 330004 Jiangxi Province China; 5 Institute of Life Science, Nanchang University, Nanchang, 330031 China

**Keywords:** Post-stroke fatigue, Stroke, Transcranial direct-current stimulation, Rehabilitation, Randomized controlled trial

## Abstract

**Introduction:**

Post-stroke fatigue (PSF) is an abnormal, persistent, and unexplained physical and psychological tiredness in patients after stroke. It is a common symptom of stroke patients with poor quality of life and bleak prognosis, and the incidence rate is up to 39% to 72%. It has been widely reported that medicine treatments achieved a lot of progress, there still needs to develop more powerful new strategies to more powerful effect. The transcranial direct-current stimulation (tDCS) shows great potential for the treatment of PSF. This study proposes to apply a double-blind randomized controlled clinical trial to explore the effect and safety of tDCS combined with routine rehabilitation for PSF.

**Methods and analysis:**

One hundred patients with PSF will be randomly divided into two groups. One of the groups will receive conventional rehabilitation therapy and active tDCS, whereas another group will receive conventional rehabilitation treatment and sham tDCS. Both groups will receive the intervention for 4 weeks, during which time they will undergo either active or sham tDCS 20 min a day, 6 days a week. Primary outcome: Fatigue Severity Scale (FSS) will be measured at baseline every weekend during the intervention period. Secondary results: Fatigue Impact Scale (FIS), Functional Assessment Chronic Illness Therapy (Fatigue) (FACIT-F), and Specialized Quality of Life Scale in Stroke (SS-QOL) will be measured at baseline and at the end of the intervention time of 4 weeks. Throughout the study, adverse events and adverse reactions will be measured during every treatment. The research study “Effects of transcranial direct current stimulation on patients with post-stroke fatigue” has been approved by the Ethics Committee of the First Affiliated Hospital of Nanchang University: Clinical Medicine Ethics Review [2015]043 in Nov 2015.

**Discussion:**

This study will provide insight into the efficacy of transcranial direct-current stimulation for post-stroke fatigue. This is a double-blind randomized controlled trial whose aim is to assess the effects of tDCS on PSF. This study can provide more information about the treatment of PSF. This study has a period of follow-up, which allows for greater accuracy. It is a single-center trial, and this may be a limitation. The other limitation of this study is the relatively small number of participants; thus, the influence of chance on experimental results cannot be completely ruled out.

**Trial registration:**

Chinese Clinical Trial Registry ChiCTR2000031120. Registered on March 22, 2020. This protocol version number is V1.1.

## Introduction

### Background and rationale

Fatigue is defined as the decrease in physical and/or mental performance that results from changes in central, psychological, and/or peripheral factors [1]. Post-stroke fatigue (PSF) is defined as observable and measurable performance degradation that occurs during the repetition of a physical or mental task. It is an early feeling of exhaustion, boredom, and aversion to effort. PSF was first mentioned in 1999 and has been evaluated separately by the medical community to distinguish it from other post-stroke psychosocial disorders. According to previous studies, 39–72% of stroke survivors have PSF.

Descriptions of fatigue include different aspects of the phenomenon, problems related to self-control and emotional instability, reduced mental capacity, and energy requirements for daily activities, such as reading and participating in physical or social activities. Fatigue can be divided into objective fatigue and subjective fatigue. And PSF is often referred to as subjective fatigue.

As for the characteristics of fatigue, PSF is qualitatively different from pre-stroke fatigue that may be exacerbated by stress and physical activity while responding well to rest, sleep, and hypothermia. The pre-stroke fatigue aggravated by physical activity is also known as exercise-induced fatigue. Exercise-induced fatigue is acute, with rapid onset, short duration, and a short recovery period. It usually occurs after vigorous physical exercise or the use of mental work. PSF, on the other hand, is chronic, long-lasting, and difficult to recover from. It can occur in the persistent activities of daily life such as taking a shower.

About the pathogenesis of PSF, repeated exhaustive exercise could result in up-regulation of the expression of mGluR1 and mGluR5 in the cortex M1 zone of rats suggesting that M1, with different time effects, contained important receptors related to the production of exercise fatigue. A transcranial direct-current stimulation (tDCS) of M1 and dorsolateral prefrontal cortex (DLPFC) significantly increased brain excitability in the M1 for at least 30 min [1]. Therefore, it suggests that we can improve fatigue by tDCS on the DLPFC.

TDCS is a noninvasive brain stimulation method that regulates cortical excitability by applying 1–2 mA direct current through the scalp. It requires at least one stimulator, electrode, and return electrode to loop. It can be divided into anode and cathode stimulation modes. By using tDCS to stimulate the motor cortex, it was found that anode stimulation can improve cortical excitability [2], whereas cathode stimulation can reduce cortical excitability. Furthermore, the effect of it is not only limited to the stimulated area, but also involves nearby brain regions [3] and can change the functional connectivity between brain regions [4]. If the time and intensity of stimulation are enough, the change of cortical excitability after a single stimulation can last for about 1 h [5] In the previous experiments [6], tDCS was found to improve fatigue using sham-controlled crossover designs, with between 10 and 25 participants and five tDCS treatment sessions using a motor, sensory, or dorsolateral prefrontal cortex (DLPFC) montage [6, 7]. The most recent study by Chalah et al. demonstrated that the DLPFC (left anodal) when compared to the posterior parietal cortex led to the highest fatigue-specific improvements.

One of the advantages of tDCS is that it can be regulated by the experimenter actively. Compared with transcranial magnetic stimulation, it is obvious that tDCS has a lower resolution. Conversely, it is less expensive, portable, and easier to use. More importantly, it was found that it has no side effects other than a slight tingling sensation.

Recently, tDCS is increasingly being used in many fields, such as dysphagia, head injuries, Alzheimer’s disease, Parkinson’s disease, acute and chronic pain, tinnitus, and depression. It is also being tested on healthy people [8]. However, few clinical studies have reported a combination of tDCS and PSF recovery.

### Objectives and hypotheses

The objectives of this randomized controlled trial are as follows:

1. To study the efficacy of tDCS in combination with conventional rehabilitation;
2. To evaluate the safety of tDCS stimulation in patients with PSF; and
3. Presentation of the research plan and the results of previous clinical trials to ensure compliance with previously recommended guidelines.

### Trial design

**Fig. 1:**
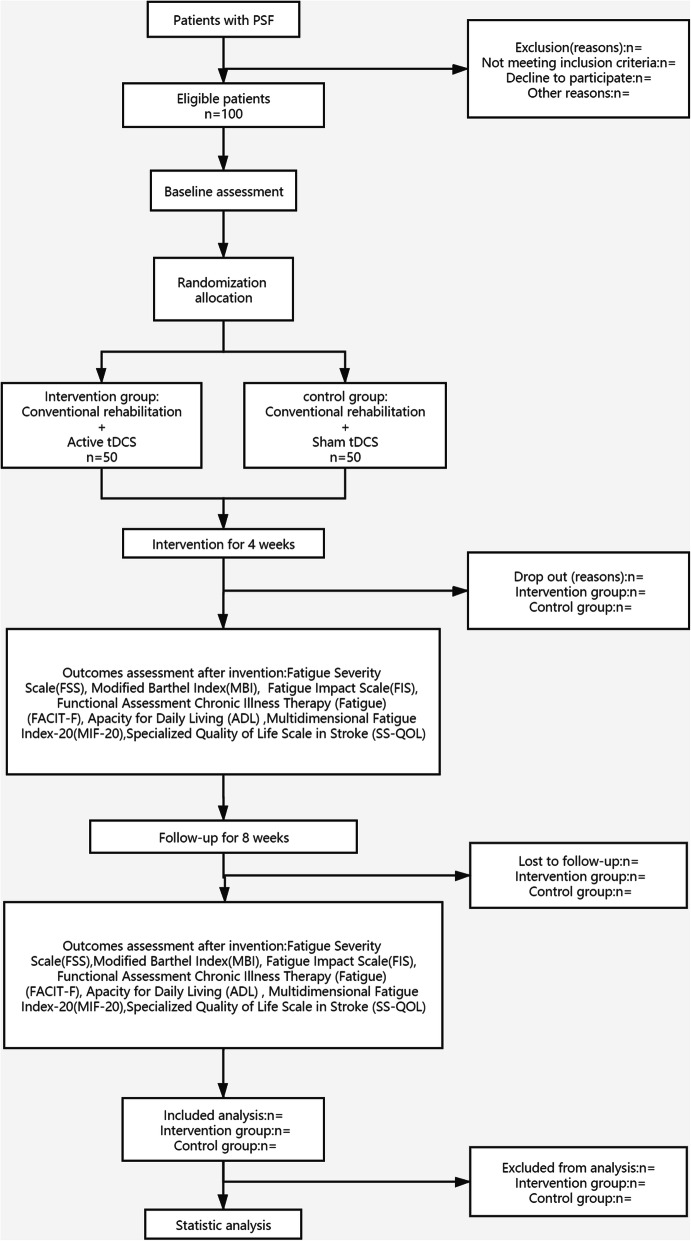
Flow diagram of the trial design. tDCS, transcranial direct current stimulation; PSF, post stroke fatigue

The clinical study is a prospective, randomized controlled trial designed with double-blinded assessments. Since there is no clinically effective treatment, we will perform the superiority test. This study will be carried out with 100 hospitalized patients with PSF. All participants will be randomly allocated to either the intervention or control group in a 1:1 ratio. Each group will, therefore, be composed of 50 patients. Interventions in both parallel groups were identical except for effective tDCS treatment. The control group will receive conventional rehabilitation combined with sham tDCS treatment, whereas the intervention group will receive conventional rehabilitation combined with active tDCS treatment. This trial will be composed of a 4-week intervention and an 8-week follow-up period. From the first to the fourth week, the primary outcome Fatigue Severity Scale (FSS) will be measured at baseline on every weekend. The secondary outcomes Fatigue Impact Scale (FIS), Functional Assessment Chronic Illness Therapy (Fatigue) (FACIT-F), and Specialized Quality of Life Scale in Stroke (SS-QOL) will be performed at the end of the intervention period. Adverse events and untoward effects will be supervised in each treatment. The first affiliated Hospital of Nanchang University (NCU), which will undertake the study, is responsible for training rehabilitation therapists on the standard operating procedure and supervising the progress of this trial at all clinical sites. In addition, the randomization and blinding will be performed by an independent statistician from the Center of Evidence Based Medicine, NCU. A flow diagram of this trial is presented in Fig. 1.

## Methods

### Study setting

Participants will be recruited from the intensive-care unit of First Affiliated Hospital. With informed consent, potential participants will be screened by neurologists at the Neonatal Intensive Care Unit of the First Affiliated Hospital of Nanchang University based on inclusion and exclusion criteria. The patients who are eligible for this study are those who meet all the requirements. Eligible individuals will be assessed by a neurologist for diagnosis and given a rehabilitation assessment. All study participants will be required to provide informed consent for themselves and their family members.

### Eligibility criteria

Patients who meet the following criteria will be considered eligible to participate in the trial:

1. Stroke appeared at least 3 months ago and within 1 year, to ensure that they are not in the acute phase of stroke.
2. Apparent fatigue, decreased energy, need for increased rest time, or fatigue out of proportion to physical activity.
3. One of the following is true (FSS average score is more than 4):

- Sleep or rest is difficult to achieve or recover,
- Motivation is retained and productivity is reduced,
- Self-perception is required to overcome this lack of vitality,
- Fatigue affects daily life/tasks,
- Fatigue lasts for several hours after exercise,
- Fatigue is a significant concern,

(4) Male or female patients aged 18–65 years.
(5) By magnetic resonance imaging (MRI), patients with no significant head displacement, structural damage, extensive necrosis of the brain structure, no significant pyramidal tract necrosis or thalamic injury in the brain stem, and not more than 30% of each lobe damaged on one side of the brain.
(6) The patient’s condition and vital signs are stable, and the patient's family voluntarily participated in the trial and signed an informed consent form.
(7) The patient has no other serious complications, such as respiratory failure, acute heart failure, severe pulmonary infection, and upper gastrointestinal bleeding.

Patients who met the following criteria would be excluded from the trial:

1. Sedatives, anesthetics, psychoactive drugs, muscle relaxants, or Na+ and Ca2 + channel blockers, such as carbamazepine, will be administered during the evaluation period.
2. The patient relies on inhalers.
3. The course of disease is more than 1 year.
4. There are any contraindications, such as pacemaker, denture, and metal prosthesis.
5. There is an epilepsy or seizure history confirmed by EEG.
6. Serious diseases such as heart, liver, and kidney failure.
7. Progressive nervous system diseases, such as the central nervous system or degenerative diseases.
8. The patient has a fever.
9. The patient has a local skin injury or inflammation.
10. The patient has hemostasis, coagulation, or anticoagulation dysfunction.
11. Acute large area cerebral infarction.
12. High sensitivity of pain stimulation area.
13. Brain injury, brain parasitosis, or brain tumor.
14. Hemiplegia or dysfunction of limbs (patients with a Fugl-Meyer scale score below 85).
15. Accompanied by aphasia.
16. Patients with incomplete clinical data and poor compliance.
17. Accompanied with serious organ disease, endocrine disease, and mental illness.
18. Patients with a score of 10 or more on the PHQ-9 scale.

### Interventions

The intervention group will receive active tDCS as well as conventional rehabilitation, whereas the control group will receive sham tDCS as well as conventional rehabilitation. Conventional rehabilitation includes basic treatments and limb electrical stimulation. The treatment will be given six times a week for 4 weeks. To improve adherence, in terms of quality management, all procedures performed by the therapists involved in the study will be standardized, including our protocols, treatment methods, and evaluations.

#### Basic treatments

Controlling intracranial pressure, blood pressure, body temperature, and blood sugar of patients will be done as basic regular treatments. Other treatments include prevention and treatment of platelet aggregation, maintenance of electrolyte and acid-base balance, prevention of complications, inchoate rehabilitation treatment for patients, regular turning over for patients, attention to the placement of limbs, daily training of joint muscles, up and down stairs, and daily life self-care ability. Depending on the patient's situation, the clinician will manage each patient, including the drug use and prevention of complications.

#### Limb functional electrical stimulation

The Functional Electrical Stimulate (FES) equipment designed at Zhongshan Memorial Hospital of Sun Yat-sen University will be used in the study. The patient will receive four-channel stimulation, performed at a frequency of 30 Hz and a pulse width of 0.2 ms [9].

FES is the application of electrical stimulation to induce muscles that have lost nerve control to contract and produce movement. Its use will not affect the comparison of the effects of TDCS in this experiment and is one of the routine rehabilitation treatment projects for dysfunction after stroke.

#### Active tDCS

The intervention group participants will be treated with active tDCS. We will place the anode of the electrode in the dorsolateral prefrontal cortex (DLPFC) on the left side of the patients’ forehead, and cathode in the superior margin of the right orbit. The current intensity will be 1.5 mA according to the previous article [10]. The diameter of the electrode plate is 5cm. The unit type is MBM-I (Jiangxi Huaheng Jingxing Medical Technology Co., LTD, Nanchang City, Jiangxi Province, China). The treatment parameters will be 20 min per session, once a day, and 6 times a week. Patients will receive tDCS treatment alone, not at the same time as other treatments. The treatment will be carried out in the neuromodulation room, at the appointed time, by a specialized therapist.

#### Sham tDCS

Participants in the control group will be treated with sham tDCS treatment. The anode of the electrodes will be located at the DLPFC on the left side of the forehead and cathode at the superior margin of the right orbit. The current will only be input every 15 s during the initial phase, and there will be no current output during the intermediate 19.5 min of sham therapy. The rest of the parameters will be the same as for active stimulation.

### Trial outcomes

#### Outcome measures

In this study, primary outcomes will be measured at baseline and at the end of every week from the 1st to the 4th week. Secondary outcomes will be measured at baseline and the end of the 4th week. During each treatment, adverse events and untoward effects will be assessed. All outcome assessments will be independently performed by experienced and blinded assessors, the same one professional rehabilitation physician. A summary of all the measures in the trial is shown in Table 1.

**Table 1:** Data on the design of the study

Point in time	Screening stage	Treatment period 1 (1 week)	Treatment period 2 (2 weeks)	Treatment period 3 (3 weeks)	Treatment period 4 (4 weeks)
Informed consent	**×**				
Inclusion and exclusion criteria	**×**				
Withdrawal, drop out, and termination criteria		**×**	**×**	**×**	**×**
Basic information	**×**				
Past medical history	**×**				
Therapeutic parameter record		**×**	**×**	**×**	**×**
**(*Baseline*) Fatigue Severity Scale (FSS)**	**×**	**×**	**×**	**×**	**×**
**Functional Assessment Chronic Illness Therapy (Fatigue) (FACIT-F)**	**×**				**×**
**Fatigue Impact Scale (FIS)**	**×**				**×**
**Specialized Quality of Life Scale in Stroke (SS-QOL)**	**×**				**×**
Overall efficacy evaluation					**×**
Complications	**×**	**×**	**×**	**×**	**×**
**Adverse events recorded**	**×**	**×**	**×**	**×**	**×**

#### Primary outcomes

##### Fatigue Severity Scale (FSS)

FSS is one of the most widely known and used scales composed of 9 items and evaluated by 7 points [11–15]. FSS points increase from 1 point (highly disagree) to 7 points (highly agree). In 1989, Krupp et al. developed the scale and applied it to patients with systemic lupus erythematosus and multiple sclerosis [16]. Aside from multiple sclerosis (MS), this scale has also been used in Parkinson’s disease, chronic fatigue syndrome, brain injury, and other diseases. The result will use the aggregate score as the patient’s score.

#### Secondary outcomes

##### Functional Assessment Chronic Illness Therapy (Fatigue) (FACIT-F)

The FACIT-F was developed in 1997 and first used to measure fatigue in oncology patients with anemia and is also a stand-alone questionnaire in the Functional Assessment in Cancer Therapy measurement system [17]. It has since been widened to include the assessment of chronic illnesses (as the FACIT measurement system). The current version of FACIT-F is the fourth. It has been used in assessing fatigue in patients with rheumatoid arthritis (RA), psoriatic arthritis (PsA), primary Sjögren’s syndrome (PSS), osteoarthritis (OA), and systemic lupus erythematosus (SLE) [18–27], as well as many other long-term conditions (e.g., multiple sclerosis, cancer, neurologic disorders).

##### Fatigue Impact Scale (FIS)

FIS is designed by Fisk to assess the impact of fatigue on quality of life. It is used to measure the impact of fatigue on patients’ physical, psychological, and cognitive functions in the first month.

##### Specialized Quality of Life Scale in Stroke (SS-QOL)

The purpose of this scale is to understand the current quality of life of stroke patients in our province and health defects after stroke. Through the analysis of the defect problem to determine the corresponding key rehabilitation content, it can help patients with stroke to maximize functional recovery and improve patient quality of life. The original SS-QOL questionnaire measures 12 domains with 49 items [28]. The domains and items were derived from interviews with stroke survivors in the USA. The validity of the SS-QOL scale has been examined in individuals after stroke in various countries, e.g., in Denmark with an ischemic stroke population [29], Nigeria (Yoruba language) [30], Mexico [31], and Germany, where a short and long version for survivors of hemorrhagic or ischemic stroke has been validated [32].

### Follow-up

After the 4-week treatment period, the patients will be followed up for 8 weeks. The medical staff will follow up with the participants by phone. Participants will be contacted every 2 weeks to record medication and rehabilitation. During the last week of follow-up (the 8th week after the intervention), participants will be referred for clinical evaluation to assess their prognosis and disability status.

### Study endpoints

The endpoints of the study are as follows:

1. In the event of a serious adverse reaction during the course of the test, the test will be terminated in time to protect the subject.
2. In the event of a serious complication or deterioration in the course of the test, the test will be terminated.
3. The test will be terminated if the subject is asked to withdraw from the clinical study.
4. And if the patient does not cooperate and does not receive treatment and the therapist’s explanation is not working, then the study will be suspended.

The researchers will keep detailed records of why and when the subjects quit the study.

### Safety assessments

The medical staff will record adverse events (AEs) that occur at any time during treatment. If a serious AE occurs, the Ethics Committee of the First Affiliated Hospital of Nanchang University will determine if the participant needs to be withdrawn from the study. We will undertake to treat AEs free of charge.

### Sample size

The sample size calculation is based on improvement in scores on the Fatigue Severity Scale (FSS). According to similar published articles [33], the FSS scores after inventions are 20.66 ± 4.54 and 40.36 ± 9.58, *n* = 57. According to the study, treatment can probably improve the FSS by 19.7 points. Improvement will be measured according to the same sample size of the estimation formula:

$$ n=2\left[{\left(\upmu \alpha +\mu \beta \right)}^2{\sigma}^2\right]/{\delta}^2 $$

With a type I error of 5% (*α* = 0.05) and 90% power (*β* = 0.10), the estimated required sample size is 43 participants per group. Allowing for a 20% dropout rate during the study, a minimum total of 100 participants is needed to reach the target of 43 participants per group.

### Recruitment

We will publish our recruitment and current results on the website of the First Affiliated Hospital of Nanchang University from January 1, 2020 to December 31, 2025. Our team will search the patient's medical records through the computer patient record system. We will select patients who are eligible for inclusion and inform the patient and his/her family of the information about the trial by phone or email. Interested families will be encouraged to contact the project manager for more information.

### Randomization and allocation concealment

In this study, randomization and allocation of hidden blocks will be used. Participants will be assigned to either the intervention or control group in a ratio of 1:1. The order of randomization will be derived using the statistical software SPSS24.0 IBM and performed by an independent statistician from the evidence-based Medicine Center NCU who will not participate in the trial. In addition, randomly assigned ratings will be hidden from the results assessors and statistical analysts. After assessing the basic information of the participants, the allocation of the eligible participants will also be concealed from their caregivers and therapists, including acupuncturists and cognitive therapists, who will be assisting patients to receive treatment.

### Blinding

Because of the double-blind implementation of this study, the “third-party” personnel who did not participate in the experiment will manage and supervise the implementation of the blind method:

1. Patients will not be allowed to open the envelopes indicating the order in which they will be involved in the study. The tDCS model is assumed to be modes A and B, and the project implementer will not know what the stimulus represents.
2. A mode is the active stimulus, and B is the sham stimulus; treatment outcomes will be assessed by third-party assessors who will not be aware of the grouping.
3. In order to prevent the analyst’s subjective tendency in the process of data analysis, the first non-blind method will be performed before the statistical analysis is completed. In other words, the analyst will know that patients are divided into two groups, but will not know which group is the intervention group. After statistical analysis, a second non-blind test will be performed to determine which group will be the intervention group.

### Statistical methods

#### Statistical analysis

Statistical methods using statistical analysis software SPSS21.0 will be used to analyze the test results. In the descriptive analysis of samples, the continuous variables are expressed in mean and standard deviation for the data of normal distribution and in the range of median and quartile for the data of non-normal distribution. The normally distributed packets are compared statistically between the *t* test groups. The ordinal level variables of the non-normal distribution are compared statistically with the Mann-Whitney *u* test. Measures with discrete distributions will be expressed as percentages and analyzed using accurate tests of either chi-square *Χ*^2^ or Fisher, as appropriate. If necessary, we will use the general linear model or logit model to adjust for confounding effects.

We will use the *t* test or the Mann-Whitney test to compare baseline characteristics between the different groups. If there is statistical significance, inequality will be treated as a confounding factor in the final efficacy analysis. In order to compare one-time or two-time results between groups, the continuous data will be analyzed by *t* test or non-parametric test, and the data will be analyzed by Pearson chi-squared or Fisher precision test. In order to control the possible confounding variables, linear models or linear regression models will be used for dependent continuous variables and dependent classified variables. The subgroup analysis of the main results will be stratified according to the sex of the participants. Analysis of variance will be used to repeat the measurement data. The analysis of primary and secondary outcomes will be based on intention-to-treat (ITT) and a per-protocol (PP) basis. The results of the ITT analysis will be compared with those of the PP analysis to determine if they are consistent. The missing data will be treated as the last carrier of observation. Adverse events will be listed and analyzed using either a chi-square test or Fisher’s precision test.

### Data collection and management

General baseline information will be collected about patients when they are sent to the hospital.

Primary outcomes will be assessed using the FSS on every weekend, and the secondary outcomes will be performed at the end of the 4th week of treatment. Complications during follow-up will also be assessed. If the patient is still in the hospital, the investigator may visit the patient on the ward to go through the evaluation. If the patient has been discharged, his or her legal representative will be told to come back in due time for assessment after leaving the hospital. If the patient does not arrive, the investigator will try to contact the patient or the family members by telephone. Other possible methods may also be used to explain the situation and complete the outcome assessment. If all attempts fail, no further contact will be made, and the patient will be recorded as lost to follow-up.

All the data will be collected by independent investigators who are blind to the patient’s allocation. Each local study center will assign a specific investigator at the beginning of the trial. This investigator will be excluded throughout the treatment of all the participants unless asked by clinicians to perform the assessment.

All variables specified in the protocol will be documented on standardized hard-copy case report forms (CRFs) in all participating centers. When the 8-week follow-up is complete, data in the CRF of each patient will be validated for completeness, consistency, and plausibility, by an independent investigating physician in a local center. Then the CRF will be transmitted to the Center of Evidence Based Medicine, NCU, which will be responsible for the development of a central database, and data entry and storage. At the end of the trial, the database will be locked and sent to the study statistician for analysis based on a predetermined statistical analysis plan.

### Data Safety and Monitoring Committee (DSMC)

The project will be monitored by the DSMC, initiated by the clinical trial center of The First Affiliated Hospital of Nanchang University, and composed of specialists in rehabilitation, ethics, statistics, and methodology. It is independent from the sponsor and has no competing interests. The Project Management Group and DSMC will audit the study through regular interviews or telephone calls once a week. Based on its review the DMC provides the sponsor with recommendations regarding study modification, continuation, or termination.

### Ethics approval and consent to participate

The research study “Effects of transcranial direct current stimulation on patients with post-stroke fatigue” has been approved by the Ethics Committee of the First Affiliated Hospital of Nanchang University: Clinical Medicine Ethics Review [2015]043 in Nov 2015. All family members of study participants will be informed of the trial details and their consent obtained by the doctors from the intensive-care unit and rehabilitation medicine department of First Affiliated Hospital. Before the trial, doctors will conduct a session to inform the participants and their families about the principles, precautions, and adverse reactions of the trial. On the consent form, participants will be asked if they agree to the use of their data should they choose to withdraw from the trial. Participants will also be asked for permission for the research team to share relevant data with people from the universities taking part in the research or from regulatory authorities, where relevant. This trial does not involve collecting biological specimens for storage. All of the relevant files are available from the corresponding author on request.

### Dissemination

The results will be published in a peer-reviewed journal 6 months after the trial ends. But no personal information about any of the participants will be disclosed.

### Patient and public involvement

This research will be carried out without any patient involvement. The patients will neither be involved in the design of the trial nor consulted to obtain or interpret the results. Patients will not be invited to contribute to the writing or editing of this document for readability or accuracy. Before publication, the results of this study will not be disseminated to patients or the public.

### Trial status

This protocol version number is V1.1. We plan to start recruiting patients for the trial on 1 December 2020, and it is expected to end on 30 June 2022. The experiment is expected to be completed by 2025.

### Data sharing

We will publish the experimental plan and research results as an article within 6 months after the trial ends and provide relevant materials for researchers who need a trial dataset. It will be published on the website of the First Affiliated Hospital of Nanchang University. There will also be a link attached if we publish our trial result in a peer-reviewed journal. The full protocol, participant-level dataset, and statistical code will be available upon email request, provided it is used only for scientific research.

## Discussion

As a new method of brain function research and treatment, tDCS has the advantage of being a painless, noninvasive, simple operation with a curative effect. As a non-invasive brain stimulation technology, tDCS can stimulate or inhibit the cerebral cortex and promote plasticity and functional reorganization of the functional areas of the cerebral cortex to encourage rehabilitation in stroke [34, 35]. Some studies show that it can help with fatigue [36]. In terms of fatigue symptoms, the stimulation sites of tDCS in clinical research include the bilateral M1 area, bilateral S1 area, and left DLPFC. Ferrucci et al. studied 23 MS patients with bilateral M1 area anode electrical stimulation, Tecchio et al. treated 10 MS patients with S1 area electrical stimulation, and the patients’ fatigue was reduced [37]. Chalah et al. compared the effects of the left DLPFC and right posterior parietal cortex (PPC) on MS fatigue. The results showed that left DLPFC improved fatigue symptoms, while right PPC reduced emotional scores. In order to ensure the authenticity of the test results, a double-blind test design will be adopted. Fatigue Severity Scale (FSS), Fatigue Impact Scale (FIS), Functional Assessment Chronic Illness Therapy (Fatigue) (FACIT-F), and Specialized Quality of Life Scale in Stroke (SS-QOL) will be used as evaluation indices to evaluate the changes of patients’ fatigue. The aim of this project is to investigate the effect of tDCS on post-stroke fatigue and find new interventions to improve PSF.

The related scale used to evaluate the fatigue degree of patients is greatly affected by the subjective factors of patients. Therefore, the experimental results are easily affected by various factors, such as the favorite degree of therapists. However, the FSS has good internal consistency, reliability, construct, and criterion validity and is sensitive to change. It has been evaluated in several conditions where it is the recommended fatigue scale [38, 39]. Thus, we can maximize the accuracy of the experiment.

In addition, because this experiment is based on a small sample, it forms an integral aspect of our future research work to provide a new medical basis for the treatment of patients with tDCS. Because of the high incidence of post-stroke fatigue, which has a great impact on the life of patients, it is particularly important to explore an effective way to treat post-stroke fatigue. This experiment on the therapeutic effect of tDCS on PSF will provide a new direction. This experiment is an active exploration for the treatment of PSF.

## Abbreviations

tDCS: Transcranial direct current stimulation
PSF: Post-stroke fatigue
DLPFC: Dorsolateral prefrontal cortex
FSS: Fatigue Severity Scale
FIS: Fatigue Impact Scale
FACIT-F: Functional Assessment Chronic Illness Therapy (Fatigue)
SS-QOL: Specialized Quality of Life Scale in Stroke
NCU: Nanchang University
FES: Functional Electrical Stimulate
MS: Multiple sclerosis
RA: Rheumatoid arthritis
PsA: Psoriatic arthritis
PSS: Primary Sjögren’s syndrome
OA: Osteoarthritis
SLE: Systemic lupus erythematosus
ITT: Intention-to-treat
PP: Per-protocol
AEs: Adverse events
PPC: Posterior parietal cortex
